# Erythema Nodosum Associated with Kerion: A Case Series and Narrative Review of the Literature

**DOI:** 10.3390/jof11020103

**Published:** 2025-01-29

**Authors:** Teerapong Rattananukrom, Isaías Uh-Sánchez, Carlos Atoche-Dieguez, Nixma Eljure, Carlos Garcia-Rementeria, Roberto Arenas

**Affiliations:** 1 Division of Dermatology, Department of Medicine, Faculty of Medicine Ramathibodi Hospital, Mahidol University, Bangkok 10400, Thailand; teerpongrattananukrom@gmail.com; 2 Servicio de Dermatología, Hospital General Dr. Adolfo López Mateos, Mexico City 01030, Mexico; uhisaias@gmail.com; 3 Centro Dermatológico de Yucatán, Mérida 97000, Mexico; atoche.d@gmail.com (C.A.-D.); dranixma@hotmail.com (N.E.); 4 Southwestern Dermatology, Oklahoma City, OK 73139, USA; cayo89_2012@yahoo.com; 5 Mycology Section, “Dr. Manuel Gea Gonzalez” General Hospital, Mexico City 14080, Mexico

**Keywords:** erythema nodosum, griseofulvin, itraconazole, kerion, scarring alopecia, tinea capitis, *Trichophyton mentagrophytes*

## Abstract

Kerion is a form of inflammatory tinea capitis, a fungal infection caused by various zoophilic, geophilic, and anthropophilic pathogens. Erythema nodosum (EN), a form of septal panniculitis, can be considered a dermatophyte id reaction that occurs outside the primary site of dermatophyte infection. The association between EN and kerion is rarely reported, with most cases following *Trichophyton mentagrophytes* scalp infections. Here, we describe three cases of EN associated with kerion caused by *T. mentagrophytes*, successfully treated with itraconazole or griseofulvin plus prednisone. Additionally, we conducted a narrative review of the literature, identifying 23 reported cases of EN associated with kerion on PubMed. The most commonly reported fungus was *T. mentagrophytes* (78.25%). In 52.17% of cases, patients developed EN after initiating antifungal treatment, with a mean onset time of 11.58 days (SD 7.3). Griseofulvin remains a mainstay treatment. The mean time for EN resolution was 8.31 days (SD 4.15), and the median duration of treatment for kerion leading to a complete response was 6 weeks (IQR 6–8). Scarring alopecia is a common sequela following kerion, and the use of corticosteroids has been recommended as adjunct therapy to minimize the risk of scarring.

## 1. Introduction

Erythema nodosum (EN), a septal panniculitis, is associated with various diseases, infections, drugs and systemic conditions. Clinically, EN is typically characterized by episodes of painful subcutaneous nodules, predominantly located on the pretibial regions, although it may also appear on the upper extremities. EN is regarded as a dermatophyte id reaction (dermatophytid), manifesting at a site distinct from the primary dermatophyte infection [1,2,3,4].

Kerion is a severe inflammatory variant of fungal infection, most frequently affecting the scalp. This condition predominantly occurs in children from rural areas and arises from zoophilic, geophilic and anthropophilic pathogens, including *Trichophyton* and *Microsporum* spp. *M. canis* is the most common causative agent in Europe, China, and South America, while *T. tonsurans* is more commonly found in North America and the UK [5,6]. Kerions are typically marked by a raised, spongy, and swollen area of skin, often accompanied by pustules, crusting, and hair loss. The condition can be painful and may lead to scarring if left untreated.

Kerion and EN are frequently linked to an intense immune response to the fungal infection. The co-occurrence of EN and kerion is infrequently reported, with only 23 cases documented in the literature available on PubMed. Most of these cases develop following scalp infections caused by *T. mentagrophytes*, often emerging after antifungal therapy, however, EN may also present before treatment begins [7]. In this report, we describe three cases kerion-associated EN due to *T. mentagrophytes*, emphasizing the variability in clinical presentation and treatment outcomes.

## 2. Materials and Methods

### 2.1. Case Series

We report three cases of EN-associated kerion caused by *T. mentagrophytes*. Species identification was performed using conventional microbiological techniques, including growth on Sabouraud chloramphenicol gentamicin agar, followed by phenotypic identification. However, a limitation of our case series is the lack of molecular identification to confirm the fungal species. Written informed consent was obtained from all patients prior to their participation in this research.

### 2.2. Review of the Literature

We conducted a narrative review of the literature using PubMed with the search terms: ‘Erythema nodosum’ AND ‘Kerion’ OR ‘(Inflammatory) Tinea Capitis’. Relevant articles published between 1948 and 2024 were screened, and only confirmed cases of EN-associated kerion were included. The review incorporated studies published in English, Spanish, Italian, and French. Data extracted included demographic characteristics, clinical presentation, time to onset of EN, fungal species involved, treatment regimens, and clinical outcomes following antifungal therapy. Diagnostic criteria for an EN-type dermatophytid reaction included the following: (I) confirmed dermatophytosis, (II) eruption occurring at a site distant from the primary fungal infection, and (III) resolution of the eruption after antifungal treatment [8]. Based on these criteria, the patient was diagnosed with EN as a dermatophytid reaction. A ‘complete response’ was defined as patients achieving full recovery after antifungal therapy. The time to develop EN was categorized as occurring either before or after the initiation of antifungal treatment.

Demographic data, baseline characteristics, clinical findings, and outcomes were analyzed using descriptive statistics. Categorical variables were presented as numbers and percentages, while continuous variables were reported as means and standard deviations (SD) or as medians and interquartile ranges (IQRs).

## 3. Results

### 3.1. Case 1

**Figure 1:**
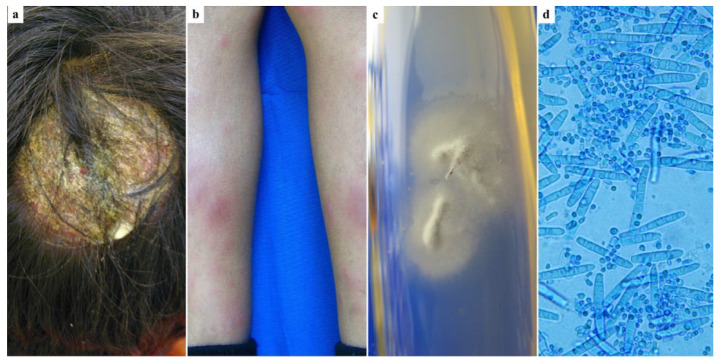
An 8-year-old boy: (**a**) Inflammatory tinea capitis on the occipital scalp; (**b**) Erythema nodosum on both shins developed prior to antifungal treatment; (**c**) Cultures of *T. mentagrophytes* showing white to creamy colonies with a cottony, mounded surface; (**d**) Microscopic features of *T. mentagrophytes* showing clustered round microconidia and cigar-shaped macroconidia. Magnification: 40×.

A healthy 8-year-old boy presented with a one-week history of painful lesions on the scalp, neck, and legs. On physical examination, a 3 cm tender, erythematous, and edematous plaque with well-defined borders and crusting was observed on the occipital region of the scalp (Figure 1a). Additionally, the patient exhibited tender cervical lymphadenopathy and multiple erythematous, painful subcutaneous nodules, measuring 1–2 cm in diameter, on both shins (Figure 1b). The patient reported no history of animal contact. A clinical diagnosis of kerion and EN was made. Potassium hydroxide (KOH) examination of the scalp lesion revealed numerous ecto-endothrix parasitizations of hairs, with a positive hair perforation test. Fungal culture showed white to cream-colored, cottony, mounded colonies characteristic of *T. mentagrophytes* (Figure 1c,d). The patient was treated with griseofulvin at a dose of 25 mg/kg/day for 30 days. His mother declined to administer prednisone due to concerns about side effects. EN resolved within one week. At the one-month follow-up, the scalp lesion had resolved, leaving only a small area of alopecia.

### 3.2. Case 2

**Figure 2:**
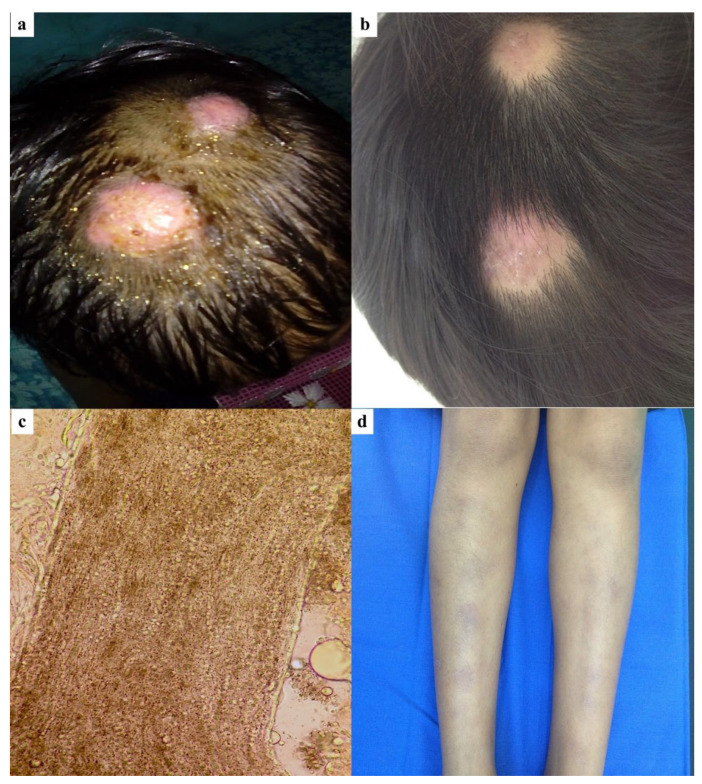
An 8-year-old girl: (**a**) Inflammatory tinea capitis on the left occipital and parietal scalp regions; (**b**) At 6-week follow-up after treatment with itraconazole and prednisone, all scalp lesions resolved, leaving focal scarring alopecia; (**c**) Ecto-endothrix parasitization in the hair shaft; (**d**) Erythema nodosum on both shins developed one week after antifungal treatment.

A healthy 8-year-old girl presented for evaluation with a 15-day history of tender, erythematous, and edematous plaques located on the left occipital and parietal regions of the scalp (Figure 2a). She denied any history of animal contact. The plaques, measuring 2 cm in diameter, displayed irregular borders, broken hairs, and crusting. KOH examination revealed hairs with ecto-endothrix parasitization (Figure 2c), with a positive hair perforation test. Fungal culture showed white to cream-colored, cottony colonies and confirmed the presence of *T. mentagrophytes*. Treatment was initiated with itraconazole at 5 mg/kg/day and prednisone at a dose of 1 mg/kg/day. During the 1-week follow-up, the patient developed multiple erythematous, painful subcutaneous nodules on both shins, suggestive of EN (Figure 2d). The treatment regimen, which included a tapering schedule for prednisone, was continued alongside itraconazole. After an additional 2 weeks, there was a 90% improvement in both the scalp and EN lesions (Figure 2b). At the 6-week follow-up, all scalp lesions had resolved, though focal areas of alopecia remained.

### 3.3. Case 3

**Figure 3:**
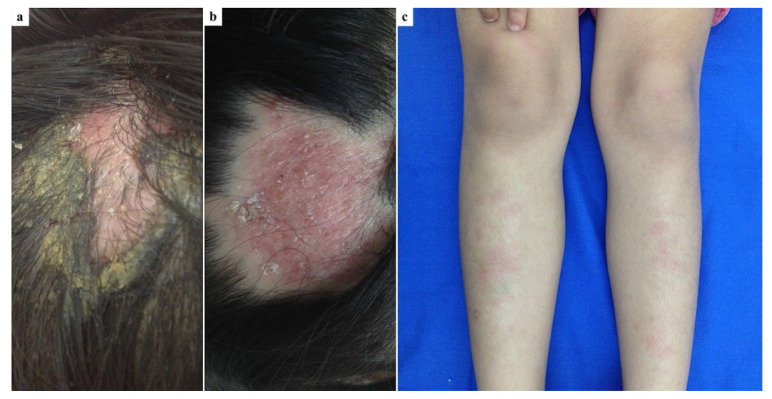
A 5-year-old girl: (**a**) Inflammatory tinea capitis on the occipital scalp; (**b**) Complete healing of the scalp lesion within six weeks, leaving a small plaque of alopecia after treatment with itraconazole and prednisone; (**c**) Erythema nodosum on both shins developed prior to antifungal treatment.

A healthy 5-year-old girl presented with a one-week history of a painful lesion on the occipital region of the scalp. The patient reported no history of animal contact. On physical examination, a tender, erythematous–edematous plaque with irregular borders and crusting was observed (Figure 3a). Additionally, the patient had multiple erythematous, painful subcutaneous nodules on both shins, suggestive of EN (Figure 3c), along with scattered erythematous, scaly plaques on the trunk and face. KOH examination of the scalp lesion revealed hairs consistent with ecto-endothrix parasitization, with a positive hair perforation test. Fungal culture showed white to cream-colored, cottony colonies characteristic of *T. mentagrophytes*. KOH examination of the facial lesions was negative for fungal elements. Treatment was initiated with oral itraconazole at 5 mg/kg/day and prednisone at 1 mg/kg/day, following a tapering schedule. At the two-week follow-up, the lesions on the trunk, face, and shins had resolved, and the scalp lesion showed complete healing within six weeks, leaving a small plaque of alopecia (Figure 3b).

### 3.4. Literature Review

A narrative review of the literature identified a total of 18 published studies, encompassing 23 cases from various countries, including Italy, Tunisia, Morocco, the UK, Spain, and the USA. The majority of cases reported before 2000 originated from Spain and the USA, whereas those published after 2000 predominantly came from Italy and Tunisia. Of the 23 cases, 16 patients were male, and 7 were female, with a median age of 8 years (IQR 6.5–9). All patients presented with either painful or painless erythematous patches or plaques, along with boggy lesions on the scalp, typically accompanied by varying degrees of pus, hair loss, and crusting, commonly referred to as “kerion” (Table 1).

The median time to onset of kerion was 14.5 days (IQR 13.75–28.5). The most common locations of kerion were the scalp (34.78%), vertex (26%), and occipital area (17.39%). Additionally, 26% of patients exhibited lymphadenitis, with cervical lymphadenitis being the most frequent type. A history of pet contact was reported in 30.43% of cases, while 17.39% of patients denied any history of pet exposure. In 52.18% of the cases, data regarding pet contact history were unavailable (Table 2).

Regarding the etiological agent, *T. mentagrophytes* was identified as the most common causative organism in 18 out of 23 cases (78.25%). Other identified agents included *M. canis* (n = 1), *Epidermophyton floccosum* (n = 1), and *T. verrucosum* (n = 1). In one case, the species could not be identified, with only mega-sporic parasitism observed. Additionally, hair direct examination was performed in only four cases, all of which revealed ecto-endothrix parasitism.

Regarding the development of EN-type dermatophytid reactions, 43.49% of patients developed EN before initiating antifungal therapy, while 52.17% developed EN after receiving antifungal treatment, with a mean onset time of 11.58 days (SD 7.3). The most common location of EN was the shins or lower legs (43.48%), followed by the legs (34.78%). Skin biopsy confirmed EN in only six cases, while the remaining cases were diagnosed based on clinical presentation and previously mentioned diagnostic criteria. Two patients exhibited not only typical EN but also erythematous papular lesions on the ears, abdomen, and back, as well as erythematous patches on the trunk, suggesting a double dermatophytid reaction. The mean healing time of EN following antifungal therapy was 8.31 days (SD 4.15).

Griseofulvin was the most commonly used antifungal agent, administered in 95.65% of cases at a dose of 10–25 mg/kg/day. Only one case was treated with terbinafine. Additionally, two patients received systemic corticosteroids (prednisolone) at a dose of 1 mg/kg/day. Topical antimycotics were added as adjunctive therapy in 43.47% of cases. The median duration of treatment for kerion and complete response was 6 weeks (IQR 6–8). All patients experienced focal scarring alopecia as a sequela.

## 4. Discussion

Erythema nodosum (EN) is the most common form of acute panniculitis, with an incidence of approximately 1 in 100,000 individuals [7]. It is more prevalent in adults, particularly affecting women between the ages of 15 and 40 years. EN is less frequently observed in children, typically between the ages of 4 and 14 years. The etiology of EN is diverse, involving a range of microorganisms and non-microbial agents, with approximately 30–50% of cases considered idiopathic.

According to the literature, the clinical manifestation of dermatophytid reactions may vary based on the host’s immunological response, presenting either in a localized or generalized form [22]. Approximately 4–5% of patients with dermatophyte infections develop a dermatophytid reaction, which can occur either concurrently with or after the primary infection. In cases of kerion, the dermatophytid reaction is commonly characterized by papules and vesicles on the face, which may subsequently spread to the trunk. Other dermatophytid-associated skin lesions include urticarial papules, follicular papules, erythema multiforme, erythema annulare centrifugum, erysipelas-like dermatitis, generalized exanthematous pustular eruption, and EN [12]. Notably, ‘the ear sign’ is a clinical feature of the id reaction observed in some cases of inflammatory tinea capitis, particularly those caused by *T. mentagrophytes.* It is characterized by redness, swelling, and pain around the ear, which may accompany typical signs of tinea capitis, such as scalp scaling, hair loss, or pustules.

We present the first case series of kerion reported in Mexico. The patients, aged between 5 and 8 years, included two girls and one boy. The disease onset ranged from 7 to 15 days. All patients exhibited kerion in the occipital region and denied any history of pet contact. One patient also had cervical lymphadenopathy. The causative agent in all cases was *T. mentagrophytes*, with ecto-endothrix parasitization observed in the hair shafts. Our findings are consistent with previous reports, which primarily involved children, with a mean age of 8 years and a median onset time of 14.5 days.

*T. mentagrophytes* was the most common pathogen associated with kerion and EN, aligning with previous literature. Notably, all cases in our series exhibited kerion with *T. mentagrophytes* as the causative agent, characterized by ecto-endothrix parasitization, typically associated with zoonotic pathogens. However, despite the absence of pet contact in our patients, *T. mentagrophytes* may have the potential for human-to-human transmission. We recommend investigating contact history in all cases to assess possible transmission routes. Another important consideration is that *T. indotineae* was first identified and described in 2020 as a distinct dermatophyte species, previously classified as a genotype variant of *T. mentagrophytes. T. indotineae* can also cause tinea capitis. However, a limitation of earlier reports is that species-specific identification methods were not used to distinguish *T. mentagrophytes* from *T. indotineae*, as these cases were studied before 2020.

In our case series, all patients presented with EN localized on the shins, which is consistent with previous reports identifying the lower extremities as the most common location for EN. One patient also exhibited EN in combination with erythematous, scaly plaques on the trunk and face, suggestive of a double dermatophytid reaction. Based on the literature review, only two cases of this type of reaction have been previously reported. One of our cases demonstrated a similar double dermatophytid reaction. Additionally, no prior studies have compared the clinical response between EN and double dermatophytid reactions, highlighting an area that warrants further investigation.

The pathogenetic mechanism of EN associated with kerion is not yet fully understood. It is thought to be a hypersensitivity reaction to fungal antigens at a site distant from the primary dermatosis. This immunological response involves the release of fungal antigens from the infection site, followed by their opsonization by antibodies and the spread of T-helper (Th)1 cells and cytokines to other areas of the body [15].

EN development appears to be a delayed hypersensitivity reaction to fungal antigens or auto-antigens triggered by the fungal infection. The correlation between the peak of inflammation and EN suggests that the massive antigenic release from the primary inflammatory response may be a key factor, supporting the hypothesis of reactive T-cells activated by this antigenic load [3,4]. In vivo studies have demonstrated delayed hypersensitivity following intradermal injections of trichophytin, while in vitro studies using leukocyte migration inhibition and lymphocyte transformation tests show that *Trichophyton* species can elicit significant cellular immune responses and delayed-type hypersensitivity reactions [23].

Llorente et al. demonstrated elevated Th1 cytokine expression in the skin nodules and peripheral blood of most patients with EN [24]. Using semiquantitative reverse transcriptase-polymerase chain reaction (RT-PCR), they examined the messenger RNA (mRNA) expression of Th 1 (interleukin [IL] 2 and interferon-γ [IFN-γ]) and TH2 (IL-4 and IL-10) cytokines in skin biopsies and peripheral blood from 11 patients with EN and 9 healthy controls. Their findings revealed increased Th1 cytokine expression in the skin lesions and peripheral blood of most patients with EN, while no cytokine expression was observed in the skin or blood of the control group.

In a mouse model, Nakamura et al. demonstrated that skin lesions induced by *T. mentagrophytes*, a zoophilic dermatophyte, are driven by a Th1 response involved in the host defense against dermatophytosis [25]. Similarly, Koga et al. [23] found increased IFN-γ production by peripheral blood mononuclear cells following trichophytin stimulation, with RT-PCR revealing IFN-γ mRNA in tinea lesions. These findings support the hypothesis that skin lesions caused by dermatophytic infections of zoophilic origin, particularly those involving *T. mentagrophytes*, are the result of a Th1 immune response. This response may also account for the associated erythema nodosum and the spontaneous regression observed in many cases.

Collectively, these findings reinforce the hypothesis that skin lesions caused by *T. mentagrophytes* infections are mediated by a Th1 response, which plays a critical role in the host’s defense against dermatophytosis.

However, there remains debate about whether EN is due to the deposition of C3 and IgM in venules of the deep dermis and adipose tissue or whether it arises from a delayed hypersensitivity reaction [1]. One hypothesis suggests an antigen-antibody reaction, as immune complex deposits have been found around venules in the septa of the hypodermis. This theory is supported by the detection of circulating immune complexes and complement activation in patients with EN. Furthermore, direct immunofluorescence studies have shown immunoglobulin deposits on the vascular walls of the septa. However, such findings are inconsistent, indicating that other mechanisms, such as type IV hypersensitivity, may also play a significant role in the development of EN [21].

EN is a rare complication associated with kerion, typically caused by *T. mentagrophytes.* Molecular techniques such as Internal Transcribed Spacer (ITS) sequencing are valuable in identifying dermatophyte strains, though no specific ITS genotype has been consistently linked to EN-complicating kerion. ITS sequencing has revealed multiple genotypes within *T. mentagrophytes*, such as Type I, II, and III ITS sequences that may exhibit varied pathogenic behavior. Further molecular research is needed to explore potential associations between specific ITS genotypes and the occurrence of EN in kerion cases, as this could enhance understanding of strain virulence and guide clinical management.

EN lesions associated with kerion commonly appear following the initiation of antifungal therapy [1]. In our case series, two patients developed EN prior to receiving griseofulvin or a combination of itraconazole and prednisolone, while one patient developed EN seven days after beginning treatment with itraconazole and prednisolone. In the literature review, the onset of EN-type dermatophytid reactions following tinea capitis varies. EN develops in 43.49% of cases before treatment and more frequently (52.17%) near the infection’s peak, with a mean onset of 11.58 days after antifungal administration. Latency periods for EN after treatment range from one to 26 days, suggesting that the pathogenesis may involve the massive release of antigens triggered by therapy.

Several authors have associated the flare of erythema nodosum (EN) with the administration of antifungal agents such as griseofulvin and terbinafine, likely due to the release of fungal antigens during therapy. This release is thought to increase immunoreactivity, leading to a heightened response to intradermal trichophytin antigen [26]. However, in some cases, EN has appeared before the administration of griseofulvin, suggesting that other mechanisms may also be involved in the pathogenesis of this dermatophytid eruption.

EN-type dermatophytid reactions typically respond rapidly to oral antifungal therapy, with griseofulvin being the most commonly used agent. The mean healing time for EN after antifungal treatment was 8.31 days. This underscores the importance of recognizing the link between EN and kerion to ensure a combined diagnosis and an effective, unified therapeutic approach. However, due to the unavailability of griseofulvin in Mexico, two of our cases were treated with itraconazole, while one patient obtained griseofulvin from another region. In our cases, EN lesions resolved within seven days of antifungal treatment, and the duration of treatment for kerion ranged from four to six weeks, with residual scalp scarring as a sequela.

The use of corticosteroids has been recommended as an adjunct therapy to minimize the risk of scarring associated with kerion. However, a randomized comparative trial found no significant difference between treatment with griseofulvin combined with oral corticosteroids and griseofulvin alone in terms of clinical outcomes [27]. We recommend systemic corticosteroid therapy in addition to antifungal treatment when the dermatophytid reaction is particularly widespread or severe. In our cases, corticosteroid therapy was added to alleviate symptoms, provide an anti-inflammatory effect, and reduce the risk of scarring alopecia caused by kerion.

The association between kerion and EN is rare, with *T. mentagrophytes* identified as the most common causative agent. To date, only 23 cases have been reported in the literature on PubMed. In most cases, EN lesions appear after the initiation of antifungal treatment. However, in two of our cases, the lesions developed before therapy, though the significance of this finding remains unclear. Treatment with oral griseofulvin or itraconazole has been highly effective, resulting in rapid resolution of lesions in all cases.

## Figures and Tables

**Table 1:** Demographic data and clinical outcomes of the literature review of cases of erythema nodosum (EN) associated with kerion.

	Number of Patients (n = 23)
Gender, n (%)	
Male	16 (69.57%)
Female	7 (30.43%)
Age, years ± IQR	8 (6.5–9)
Onset of kerion, days ± IQR	14.5 (13.75–28.5)
Location of kerion, n (%)	
Scalp	8 (34.78%)
Vertex	6 (26.08%)
Occipital area	4 (17.39%)
Occipitoparietal area	2 (8.7%)
Parietal area	2 (8.7%)
Temporal area	1 (4.35%)
Lymphadenopathy, n (%)	6 (26.08%)
History of contact animals, n (%)	7 (30.43%)
Culture of pathogen, n (%)	
*Trichophyton mentagrophytes*	18 (78.25%)
*Microsporum canis*	1 (4.35%)
*Epidermophyton floccosum*	1 (4.35%)
*Trichophyton verrucosum*	1 (4.35%)
*T. mentagrophytes complex*	1 (4.35%)
*Negative* (seen mega sporic parasitism)	1 (4.35%)
EN location, n (%)	
Shins or lower legs	10 (43.48%)
Legs	8 (34.78%)
Shins and thighs	3 (13.04%)
Legs and forearms	1 (4.35%)
Pretibial area	1 (4.35%)
Dermatophytid (id) reaction, n (%)	
EN	21 (91.3%)
EN + other id reaction	2 (8.7%)
EN develop before receiving antifungal treatment	10 (43.49%)
EN developed after initiating antifungal treatment	12 (52.17%)
Time from treatment to onset EN, days ± SD	11.58 (7.3%)
Treatment, n (%)	
Griseofulvin	20 (86.95%)
Terbinafine	1 (4.35%)
Griseofulvin and systemic steroids	2 (8.7%)
Healing time of EN, days ± SD	8.31 (4.15)
Treatment duration for kerion, weeks ± IQR	6 (6–8)

**Table 2:** Literature review of cases of erythema nodosum (EN) associated with kerion.

Authors/Year	Country	Gender/Age (Years)	Onset of Kerion (Days)	Kerion Location	History of Contact Animals	Culture	EN Location/Other Id Reaction	Time in Days from Treatment to Onset EN	Treatment	Healing Time of EN (Days)	Treatment Duration for Kerion (Weeks)
Our series	Mexico	M/8	7	Occipital scalp + cervical lymphadenopathy	No	*Trichophyton mentagrophytes ^^^*	Shins	Before treatment	Griseofulvin 25 MKD	7	4
		F/8	15	Occipitoparietal scalp	No	*Trichophyton mentagrophytes ^^^*	Shins	7	Itraconazole 5 MKD + prednisolone 1 MKD	7	6
		F/5	7	Occipital scalp	No	*Trichophyton mentagrophytes ^^^*	Shins/Erythmatous scaly plaques on trunk and face	Before treatment	Itraconazole 5 MKD + prednisolone 1 MKD	7	6
Herzum et al. 2024 [9]	Italy	M/7	30	Scalp + occipital lymphadinopathy	No	*Microsporum canis*	Legs	Before treatment	Griseofulvin 20 MKD	NA	8
Salah et al. 2021 [10]	Tunisia	M/14	NA	Vertex	NA	*Trichophyton mentagrophytes*	Legs	14	Griseofulvin	1	NA
		M/9	NA	Vertex	NA	*Trichophyton mentagrophytes*	Legs	7			
		M/4	NA	Vertex	NA	*Trichophyton mentagrophytes*	Legs	20			
Romano et al. 2016 [2]	Italy	F/4	13	Scalp + cervical lymphadenopathy	Cat	*Trichophyton mentagrophytes ^^^*	Lower legs/Erythematous papular lesions on ears, abdomen and back	2	Griseofulvin 20 MKD + oral betamethasone	9	8
Kelati et al. 2016 [11]	Morroco	M/9	15	Vertex	Dogs	*Trichophyton mentagrophytes*	Legs, forearms *	Before treatment	Griseofulvin 25 MKD	NA	6
Mohamed et al. 2016 [7]	Tunisia	M/7	14	Temporal scalp	Rabbit	*Trichophyton mentagrophytes*	Legs *	Before treatment	Griseofulvin 20 MKD + topical econazole + mefenamic acid 500 mg/day	7	6
Castriota et al. 2013 [12]	Italy	F/9	48	Occipital scalp + Retronuchal lymphadenopathy	Rabbit, dogs	*Trichophyton mentagrophytes ^^^*	Legs *	14	Griseofulvin 25 MKD + topical mupirocin and tioconazole + prednisone 1 MKD	10	6
Zaraa et al. 2012 [1]	Tunisia	M/7	60	Occipitopariental scalp + cervical lymphadenopathy	No	*Mega sporic parasitism ^^^*	Shins, thighs */Erythematous patches on trunk	18	Griseofulvin 25 MKD + ciclopirox olamine cream	7	12
Bassi et al. 2009 [13]	UK	F/8	8	Vertex + cervical lymphadenopathy	Pet rats	*Trichophyton mentagrophytes*	Shins, thighs	1	Griseofulvin 10 MKD	10	6
Morrone et al. 2011 [14]	Italy (Philippine nationality)	F/35	21	Vertex ^^^	No	*Trichophyton mentagrophytes ^^^*	Pretibial area *	Before treatment	Terbinafine 250 mg/day + naproxen 1 g/day	15	8
Soria et al. 2008 [15]	Spain	M/11	NA	Parietal scalp	NA	*Trichophyton mentagrophytes*	Lower legs	26	Griseofulvin + Ibuprofen	7	NA
		M/9	NA	Scalp	NA	*Trichophyton mentagrophytes*	Lower legs	16	Griseofulvin	7	NA
Provini et al. 2003 [16]	Italy	M/3	56	Scalp	NA	*Epidermophyton floccosum*	Lower legs	Before treatment	Griseolfuvin+ topical eosine and myconazole	NA	NA
Calista et al. 2001 [8]	Italy	F/5	14	Right parietal scalp	No	*Trichophyton mentagrophytes*	Shins, thighs	Before treatment	Griseofulvin 18 MKD + topical crystal violet	10	6
De las Heras et al. 1991 [17]	Spain	M/9	7	Scalp	Rabbitsdogs	*Trichophyton mentagrophytes*	Shins *	Before treatment	Griseofulvin 10 MKD + topical tioconazole	12	6
Martinez-Roig et al. 1982 [18]	Spain	M/8	NA	Occipital scalp	NA	*Trichophyton mentagrophytes*	Shins	7	Griseofulvin + topical potassium permanganate solution	12	NA
		M/6	NA	Occipital scalp	NA	*Trichophyton mentagrophytes*	Shins	7		12	NA
		M/3	NA	Occipital scalp	NA	*Trichophyton mentagrophytes*	Shins	7		12	NA
Stocker et al. 1977 [19]	USA	F/12	NA	Scalp	NA	*Trichophyton verrucosum*	Shins	Before treatment	Griseofulvin	NA	NA
Velasco et al. 1975 [20]	Spain	M/7	NA	Scalp	NA	*Trichophyton mentagrophytes*	Legs	NA	Griseofulvin	NA	NA
Smith et al. 1963 [21]	UK	M/7	14	Occipitopariental scalp + cervical adenitis	White mouse	*Trichophyton mentagrophytes*	Lower legs	Before treatment	Griseofulvin + topical tioconazole	NA	NA

M, male; F, female; ^^^ ectoendothrix hair parasitism; * erythema nodosum biopsy-proven; NA, no available data; MKD, mg/kg/day.

## Data Availability

The original contributions presented in the study are included in the article, further inquiries can be directed to the corresponding author.
